# On Fermentation as a Cause of Various Diseases

**Published:** 1864-01

**Authors:** 


### ON FERMENTATION AS A CAUSE OF VARIOUS DISEASES.

M. Polli, of Milan, has recently published two very interesting memoirs on fermentation as a cause of various diseases, from which we extract some of the more important facts.

Chemists who have, of late years, investigated with the greatest success the phenomena of fermentation, have observed that this mode of reaction amongst organic principles has a much greater importance than was suspected. It is, in fact, to fermentation that the spontaneous decomposition of animal and vegetable tissues is due, such as gangrene, dry rot, eremacausis, etc., and the whole series of successive transformations that orgaric substances undergo until they are converted into water, carbonic acid, ammonia and mineral matters. It is by fermentation that fatty bodies give glycerine; that salicine furnishes glucose; that myronate of potash is converted into essential oil of mustard; that neutral substances, such as urea and allantoin, form ammonia; that amygdaline produces the poisonous substances, oil of bitter almonds and hodrocyanic acid.

Ferments act by contact or by catalysis. Sometimes they are living creatures, sometimes very active substances which are not organized. Diastase, emulsine and pepsine perform the part of ferments. They may cause organic substances to double, become hydrated or isomeric.

According to M. Polli, there exists a great analogy between the processes of fermentation and many organic metamorphoses which occur in some diseases. An albuminous matter which in a particular state of alteration acts as a ferment, and particular substances which proceed from its action; this is the basis of the humoral theory.

But analogy is not sufficient of itself; it has been shown by carefully conducted experiments that the blood in disease undergoes alterations and variations in its composition, and that artificial disease may be produced bearing a strong resemblance to natural disease, by injecting into the blood-vessels substances which act as ferments. Multiple abscesses, induced by the injection of pus into the veins of dogs; septic affections caused by injecting purulent putrid matters into the veins of animals; diseases presenting all the characteristics of typhoid fever, and caused by the injection of putrified blood into the circulating current; finally, contagious diseases, such as glanders, which is produced by the injection of glanderous humors, are facts which prove that a general affection may be induced by the simple introduction into the blood of a substance capable of acting as a ferment. The diseases which may be called catalytic, in which the morbific matter produces metamorphoses by contact with the alterable principles of the blood are the primary cause of all the symptoms presented by the animal economy. In short, it is impossible to deny that fermentation may be produced in the blood.

But admitting that the starting point of many diseases is the action of a specific ferment in the blood, is it possible to prevent its effects, to render it inactive in the living organism, as we may do apart from the body, by many chemical means? This is the great point which gives interest to this pathological question.

M. Polli believes that he has proved, by a series of facts and conclusive experiments, that it is possible to neutralize morbific ferments in the blood of animals by chemical substances which do not act in a manner incompatible with life; and it is by these substances that we must hope to treat successfully those diseases of which fermentation is the primary cause.

It is well known that sulphurous acid gas prevents alcoholic and acetic fermentation, and also the fermentation of animal substances and organic matters in general. Thus it arrests, if it be already begun, the fermentation produced by saliva and diastase in contact with starch, the fermentation which myrosine produces in the paste of black mustard flour, that which is produced by emulsine on the amygdaline of bitter almonds, etc.

M. Polli has proved that alkaline or earthy sulphites possess the same antiseptic and decolorizing properties. This is a very important fact, since it admits of the application of sulphurous acid in therapeutics. He thinks also, that he has ascertained that the action of sulphurous acid and of sulphites on coloring matters as well as on ferments, is neither a deoxygenation, a combination, nor a destruction, but simply a molecular modification.

This mode of action of sulphurous acid and sulphites explains the valuable property which these chemical compounds possess of preventing or energetically arresting the action of morbific ferments artificially introduced into the blood of animals, without altering its composition in such a manner as to be incompatible with life.

From a great number of experiments made upon dogs, and alluded to in his memoirs, M. Polli has determined the safe and efficacious dose of sulphites for internal administration, the changes which they undergo in the organization, and their curative action in the affections produced by the injection of putrid or contagious matters into the blood.

The following are some of his experiments, selected from those of the last-mentioned series:

1. Ten grammes of sulphate of soda were given to a dog during a period of five days, then one gramme of pus was injected into the femoral vein. The animal became dull, and refused the food which it was offered, but the next day its spirits returned and it ate willingly. Two days after, the same experiment was repeated and was followed by the same results. At the end of a few days the animal was perfectly cured.

2. One gramme of pus was injected in two portions into the veins of a dog of a more robust nature than that operated upon in the preceding experiment. The animal became spiritless, but the next day took some food; the following day it was very low, it breathed with difficulty, its wounds were sanious, its leg and foot swollen, and it died ten days afterwards.

3. An equal quantity of putrid blood was injected into the veins of three dogs; one died five hours after the infection, another after five days of illness, and the third, to which some sulphate of soda had been administered, after having experienced some trifling symptoms of illness, rapidly recovered.

4. Numerous other experiments made with putrid blood and morbific mucus proved that the animals died with all the symptoms of a general infection, whenever sulphite of soda was not administered, and that, on the contrary, they speedily recovered under its influence.

If these facts should be confirmed by other experiments, M. Polli will have rendered an inestimable service to therapeutics, and will have thrown some light on the yet obscure cause of numerous diseases.—*Pharm. Jour. and Trans.*, London, April, 1863, from *Journ. de Pharm. et de Chimie.*

### NITROUS OXIDE AS AN ANÆSTHETIC.

The attention of the profession has been directed within the last few months, to this agent for the production of anæsthesia. It is claimed by those who have experimented with it, that it is very efficient, operating rapidly, almost as much so as chloroform, and with perfect safety; no serious accident yet having occurred from its use. Patients are as easily controled while under its influence as any other anæsthetic agent.

The manner of its action we conceive to be very different from that of chloroform or ether; during the administration of the latter, the tendency is to deprive the blood of the ordinary amount of oxygen, while the former increases it. While death even *might* ensue from asphyxia under its full influence, yet its operation upon the nervous system, and especially upon the nerve centres, is certainly attended with far less danger than chloroform or ether.

The manner of administering it seems to constitute the greatest objection to its general use. With appropriate apparatus it is easily prepared, but it requires large space; three to six gallons being required for each case. The apparatus and mode of administration are being simplified, and it is presumable that this will be so far accomplished, that there will be no special objection in this direction. All the apparatus, with instructions for using it, is furnished by parties in New York, and other points. We hope all who have opportunity will experiment and report. T.

### SUGAR AS AN ANTHELMINTIC.

M. Debout says that sugar is an excellent destroyer of worms. He once accidentally put sugar instead of salt on a leech which he wished to detach from the skin, and was surprised at the spasms produced by it. He therefore tried sugar on earth-worms, and found it had a similar powerful effect; and has since used it in solution with success as an injection in children.—*British Med. Journal.*

